# Distribution of *Plasmodium vivaxpvdhfr* and *pvdhps* alleles and their association with sulfadoxine–pyrimethamine treatment outcomes in Indonesia

**DOI:** 10.1186/s12936-015-0903-0

**Published:** 2015-09-22

**Authors:** Puji B. S. Asih, Sylvia S. Marantina, Rodiah Nababan, Neil F. Lobo, Ismail E. Rozi, Wajio Sumarto, Rita M. Dewi, Sekar Tuti, Ahmad S. Taufik, Mulyanto, Robert W. Sauerwein, Din Syafruddin

**Affiliations:** Eijkman Institute for Molecular Biology, Jalan Diponegoro 69, Jakarta, 10430 Indonesia; Eck Institute for Global Health, University of Notre Dame, Notre Dame, IN USA; Department of Biomedicine and Pharmacology, National Institute for Health Research and Development, Jakarta, Indonesia; Immunobiology Laboratory, School of Medicine, University of Mataram, Mataram, Indonesia; West Nusa Tenggara Hepatitis Laboratory, Mataram, Indonesia; Department of Medical Microbiology, Radboud University Nijmegen Medical Centre, Nijmegen, The Netherlands; Department of Parasitology, Faculty of Medicine, Hasanuddin University, Makasar, Indonesia

**Keywords:** Drug resistance, Sulfadoxine–pyrimethamine (SP), Molecular markers, *Plasmodium vivax*, Indonesia

## Abstract

**Background:**

Sympatric existence of *Plasmodium falciparum* and *Plasmodium vivax*, and the practice of malaria treatment without microscopic confirmation suggest that the accidental treatment of vivax malaria with sulfadoxine–pyrimethamine (SP) is common.

**Methods:**

In this study, the frequency distribution of alleles associated with SP resistance were analysed among the *P. vivax* infections from malariometric surveys and its association with SP treatment failure in clinical studies in Indonesia. The *dhfr* and *dhps* alleles were detected using PCR–RFLP method.

**Results:**

Analysis of 159 *P. vivax* isolates from malariometric surveys and 69 samples from in vivo SP efficacy study revealed various the existence of various alleles of the *pvdhfr* and *pfdhps* genes including 57L/I, 58R, 61M, and 117N/T. Allele 13L of the *dhfr* gene and 553G of the *dhps* gene were not detected in any isolates examined in both studies. In the *dhfr* gene, tandem repeat type-A was the major tandem repeat observed in any isolates analysed. In the *dhps* gene, only the 383G allele was observed. Isolates carrying double, triple and quadruple mutants of *dhfr* gene were found in Lampung, Purworejo, Sumba, and Papua. Although this study revealed a wide distribution of *dhfr* and *dhps* alleles among the *P. vivax* isolates across a broad geographic regions in Indonesia, impact on SP efficacy was not observed in Sumba.

**Conclusion:**

With proper malaria diagnosis, SP may still be used as a rational anti-malarial drug either as a single prescription or in combination with artemisinin.

## Background

Sulfadoxine–pyrimethamine (SP), or Fansidar, a combination anti-malarial drug containing the sulfonamide antibiotic, sulfadoxine and the antiprotozoal pyrimethamine, has long been used as an anti-malarial drug throughout the world. Its easy single-dose prescription and relative efficacy has made this combination an excellent choice in the treatment of uncomplicated falciparum malaria and intermittent presumptive treatment in pregnancy (IPTp) in Africa with the advent of chloroquine resistance [1]. Nevertheless, the growing parasite resistance to this drug has limited its use in many parts of the world, including Indonesia.

The molecular basis of parasite resistance to SP has been established in *Plasmodium falciparum* and rodent *Plasmodium*, and various single nucleotide polymorphisms (SNPs) in dihydropteroate synthase (*dhps*) and dihydrofolate reductase (*dhfr*) genes have been linked to the resistance [2, 3]. The SP combination has never been recommended to treat vivax malaria in Indonesia, however, the sympatric existence of *P. falciparum* and *Plasmodium vivax* and the practice of malaria treatment without microscopic confirmation suggest that accidental treatment of vivax malaria with SP has often taken place. Treatment of patients with SP has inadvertently led to the simultaneous selection of SP-resistant *P. vivax.* In Indonesia before 2004, SP was used alone as a second-line anti-malarial drug for falciparum malaria [4]. Although SP has never been recommended for the treatment of patients with *P. vivax* malaria, the selection pressure exerted by the drug is expected to have continued progressively in *P. falciparum* and *P. vivax* [5]. Since 2010, vivax malaria cases in Indonesia are treated with artemisinin-based combination therapy (ACT) [6].

Pyrimethamine inhibits the *dhfr* enzyme [7] and sulfadoxine targets the *dhps* enzyme in the folate biosynthetic pathway of the parasite [8]. Point mutations in parasite *dhfr* and *dhps* genes confer resistance to SP in *P. falciparum.* High level resistance to pyrimethamine in *P. falciparum* results from the accumulation of mutations in *pfdhfr* principally at codons 16, 51, 59, 108, and 164 [9, 10]. These mutations have been shown to alter the pyrimethamine binding sites in *pfdhfr* and reduce enzyme drug interaction [11]. Twenty non-synonymous mutations have already been described in the *pvdhfr* gene [5, 12]. Some of these mutations (at codon 57, 58, 61, 117, and 173) are involved in resistance to pyrimethamine [13, 14]. Five mutations have already been identified in the *pvdhps* gene, at codon 382, 383, 512, 553, and 585, corresponding to position 436, 437, 540, 581, and 613 of the homologues gene in the *P. falciparum* [3, 14, 15]. The *pvdhfr* and *pvdhps* genotypes might be associated with treatment failure in individual vivax malaria patients [16]. Limited data are available about polymorphisms in *pvdhfr* and *pvdhps* genes of malaria parasites from Indonesia. Previous data from Lampung show triple mutation found in this area [17] and a quadruple mutant 49R/57L/58R/61M/117T was found in Papua [17, 18].

The extent of genetic polymorphisms associated with resistance to SP was screened among the *P. vivax* isolates in Indonesia towards evaluating the use of SP for the treatment of *P. vivax* malaria and the possible use of SP for IPTp.

## Methods

*Plasmodium vivax* isolates were obtained from two different studies: (1) malariometric surveys in five malaria-endemic areas; and, (2) in vivo SP efficacy study in Southwest Sumba District.

### Malariometric survey (sample set 1)

**Fig. 1:**
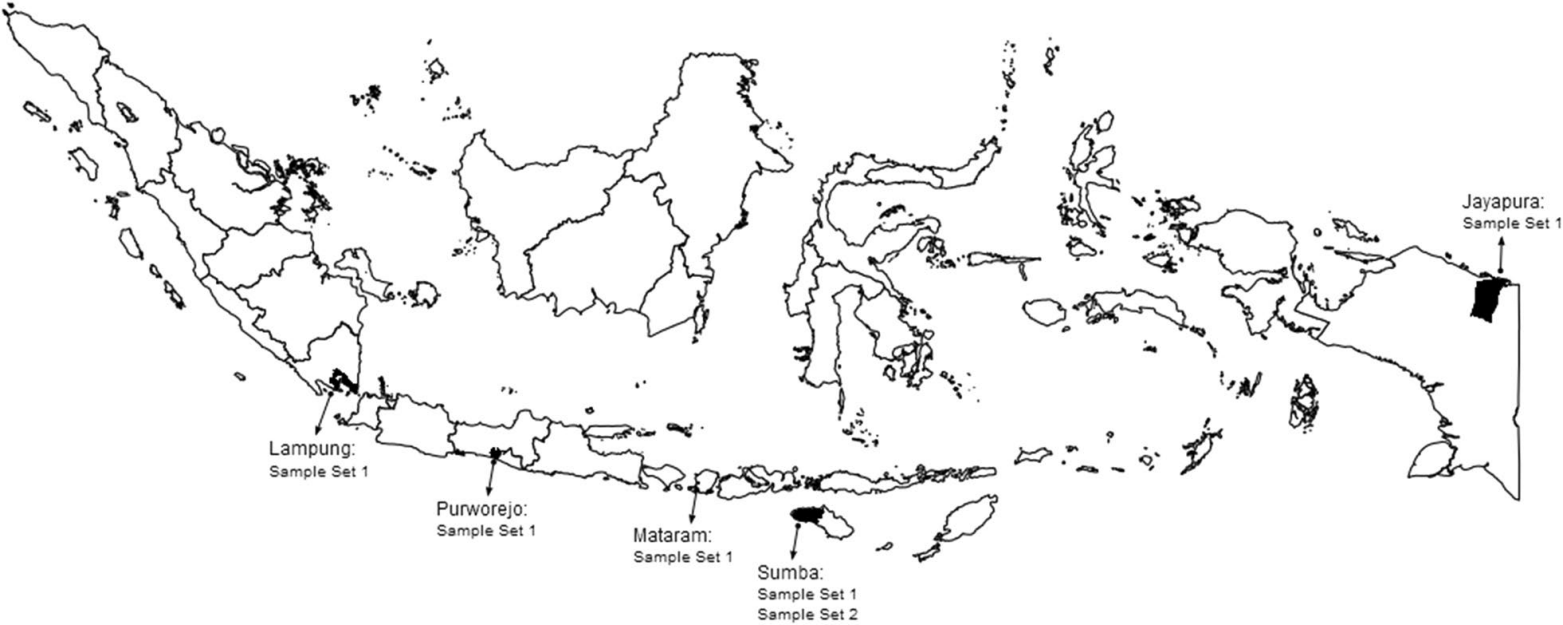
The origin of *Plasmodium vivax* isolate for sample sets 1 and 2. (1) Lampung, south of Sumatera; (2) Purworejo, Central Java; (3) Mataram, West Nusa Tenggara; (4) Sumba (East Nusa Tenggara); and, (5) Jayapura, Papua

Blood blots on filter paper (3 MM; Whatman, Hillsboro, OR, USA) containing approximately 50 µL blood equivalent were collected from five selected malaria-endemic areas: Lampung, Purworejo, Mataram, Sumba, and Papua (September 2006–August 2008) (Fig. 1).

### In vivo sulfadoxine–pyrimethamine efficacy study (sample set 2)

The study was conducted in Southwest Sumba District, East Nusa Tenggara Province from September 2009 to February 2010 (Fig. 1). The subjects were recruited from a pool of malaria-infected individuals identified during an active malariometric survey. Subjects were excluded if they met any of the following exclusion criteria: (1) were pregnant; (2) history of allergy to the study drugs or study drugs’ derivative; (3) completed treatment with an anti-malarial drug in the preceding 2 weeks; or, (4) medical history of untreated hypertension or chronic heart, kidney or liver disease. *Plasmodium vivax*-infected subjects did not show any signs of severe malaria. All study subjects were supervised in their treatment with SP tablets (30 mg sulfadoxine and 1.5 mg pyrimethamine per kg body weight, according to national treatment guidelines) and followed for 28 days. A study nurse gave medication, observed and recorded all treatments, and repeated the treatment if vomiting occurred within 30 min of the administered dose. Parasitological responses were classified according to criteria set by WHO [19]. At enrolment, a finger prick was performed to make thick and thin blood smears, blots on filter paper (Whatman, Schleicher and Schuell, Whatman International Ltd, Maidstone, UK) for further parasite genotyping, and haemoglobin measurement using hemocue (HemocueTM Hb201+, Angelholm, Sweden). Blood smears and filter-paper blood samples were also collected from finger pricks on days 1, 2, 3, 7, 14, 21, and 28. Smears were read by expert microscopists and confirmed by polymerase chain reaction (PCR). Adverse effects observed during the study were recorded by the study nurse and/or physician. Primaquine therapy against relapse was not provided until discontinuation from the study, i.e., day of recurrence or day 28. This study has been approved by the Eijkman Institute Research Ethics Committee for the use of human subjects.

### Malaria microscopy

All blood slide thick and thin blood smears were stained with Giemsa, and subsequently examined by light microscopy. Parasite density was determined by counting the number of parasites per leukocytes in 100-high-power microscopic fields in a Giemsa-stained thick film, assuming an average of 20 leukocytes/microscopic field and 8000 leukocytes/µl blood. The total number of parasites/µl was multiplied by 40 [20].

### Genomic DNA preparation

DNA from all samples (including sample set 2, on day of enrolment and day of recurrence) were extracted from the blood samples using Chelex-100 ion exchanger (Biorad Laboratories, Hercules, CA, USA) [21]. The filter papers were placed in a microtube, 100 µl distilled water and 50 µl 20 % Chelex-100 in distilled water were added. DNA was extracted by boiling at 100 °C for 10 min. The extracted DNA was either used immediately for PCR assays or stored at −20 °C for later analysis.

### Confirmation of *Plasmodiumvivax* species

A nested PCR diagnosis was performed using the 18S rRNA gene diagnostic [22] in both sets of sample isolates.

### Amplification and analysis of *pvdhfr* and *pvdhps* gene mutation

The nested PCR amplification strategies for *pvdhfr* and *pvdhps* genes were designed using previously published results [5, 23]. Two fragments were obtained by nested-PCR: one being used for the analysis of F57L/I, S58R, T61M, and S117N/T and the other for the examination of I13L, F57L/I, S58R, and T61M alleles. Both *pvdhfr* gene tandem repeat variants [7, 24] were observed and designated as type A (three tandem repeat GGDN TS GGDN TH GGDN) and type B (two tandem repeat GGDN TS GGDN and deletion at 98–103 residue). The amplified fragment of the *pvdhps* gene corresponds to GenBank acc no. AY186730 and detects mutations A383G and A553G [23].

## Results

All *Plasmodium* infection isolates were confirmed as *P. vivax* by PCR diagnosis, performed using 18S rRNA gene primer. The analysis of these *P. vivax* isolates indicated that several isolates carried mutant alleles of the *Pvdhfr*, such as 57L/I, 58R, 61M, and 117N/T and allele 383G of the *Pvdhps* genes.

### Malariometric survey (sample set 1)

#### Mutant alleles of the *dhfr* gene

Of the 159 isolates screened for *pvdhfr* mutations from five different malaria endemic areas, 131 were found to carry at least one of the *pvdhfr* mutant alleles and 28 isolates (21 %) carried no mutant allele. The allele with the highest prevalence was 117N (27 %), followed by 117T (18 %). Isolates carried the double mutant (58R/117N) were found in three sites: Papua, Lampung and Purworejo, with the highest prevalence observed in Lampung (23 %). *Plasmodium vivax* isolates with a double mutant *dhfr* allele were not found in Sumba and Mataram. The triple (58R/61M/117T) and quadruple (57L/58R/61M/117T) mutant alleles were found with prevalences of 3 and 18 %, respectively (Table 1). Mutations at codon I13L were not detected in any of the samples analysed. Three types of allelic combination of the *dhfr* gene were identified from Papua, while two types were identified from Lampung and Purworejo. One type of allelic combination was found from Sumba. Tandem repeat type A was the major tandem repeat observed among the *P. vivax* isolates in all sites. The frequency distribution of tandem repeat A ranged from 66.7 to 100 % (Table 1).

**Table 1:** Prevalence of allelic combinations and repetitive motifs in *dhfr* gene among *Plasmodium vivax* isolates in Indonesia

Type	Prevalence in malaria-endemic areas (%)				
	Papua	Lampung	Purworejo	Sumba	Mataram
Allelic combination					
58R/117N	11.8 (4/34)	96.3 (26/27)	60 (6/10)	0 (0/60)	0 (0/28)
58R/61M/117N	2.9 (1/34)	11.1 (3/27)	0 (0/10)	0 (0/60)	0 (0/28)
57L/58R/61M/117T	61.8 (21/34)	0 (0/27)	10 (1/10)	3.3 (2.60)	0 (0/28)
Repetitive motifs GGDN (units)					
Type A = three units	100 (34/34)	66.7 (18/27)	100 (10/10)	85.7 (24/28)	92.9 (26/28)
Type B = two units	0 (0/34)	33.3 (9/27)	0 (0/10)	14.3 (4/28)	7.1 (2/28)

#### Mutant alleles of the *dhps* gene

Analysis of the 159 *P. vivax* isolates revealed only one mutant allele of the *dhps* gene, 383G, in three sites with prevalence of 33, 15 and 9 % in Papua, Purworejo and Sumba, respectively. Mutant allele 553G was not observed in any of the isolates examined.

### In vivo sulfadoxine–pyrimethamine efficacy study (sample set 2)

Of the 500 subjects screened, 69 subjects met the inclusion criteria and were recruited for this study. Characteristic of the subject is shown in Table 2. Sixty-three (94 %) subjects were successfully cleared their parasitaemia before day 7 and completely recovered up to day 28 (Table 3). One subject (1.5 %) was found to carry parasites at day 14. Three subjects were lost to follow-up and two subjects dropped out (Table 3). No severe side effects were noted in any of the 69 subjects enrolled.

**Table 2:** Characteristics of the subjects at enrolment in the 28 days in vivo sulfadoxine–pyrimethamine test in Sumba

Number of subjects	69
Median (range) age (year)	13.2 (2–60)
Median (range) haemoglobin level (day 0) (g/dl)	13.2 (9.4–16.2)
Geometric mean asexual parasitaemia/µl blood	893 (40–19,600)

**Table 3:** *Plasmodium vivax* phenotyping sulfadoxine–pyrimethamine in Sumba

Total subject	ETF	LTF	ACPR	LTFU	Drop-out
69	0	1^a^	63	3	2

*ETF* early treatment failure, *LTF* late treatment failure, *ACPR* adequate clinical parasitological response, *LTFU* lost to follow-up

^a^Day 14

### Mutant alleles of the *pvdhfr* gene

Various types of allelic combination of *dhfr* gene mutants were identified from in vivo SP efficacy study in Sumba (Table 4). Analysis of the *pvdhfr* gene of the 69 subjects at day 0 and one subject at day 14 revealed the existence of four mutant alleles: 57L/I, 58R, 61M, and 117N/T, either as a single mutation or in combination with the other alleles. A single mutation at codon 57L, 61M, 117N, and 117T was found with frequencies of 1.4, 8.6, 2.8, and 1.4 %, respectively. Allele I13L was not detected in any of the isolates examined. Double mutants with four types of allelic combination (58R/61M, 58R/117N, 61M/117T, 61M/117N) were found in 24 isolates (34.3 %) including the recurrent isolate at day 14. Triple mutants with five types of allelic combination (58R/61M/117T, 57L/58R/61M, 58R/61M/117N, 57L/61M/117T, and 57L/58R/117N) were observed in 27 isolates (38.6 %). The quadruple mutant, 57L/58R/61M/117T, was found in one isolate (1.4 %). Tandem repeat type A was also the major tandem repeat observed in isolates analysed, including the recurrent isolate at day 14.

**Table 4:** Allelic combination in *dhfr* and *dhps* genes among the sulfadoxine–pyrimethamine efficacy samples

Type of allelic combination					Total isolate
F57I/L	S58R	T61M	S117T/N	A383G	
F	R	M	S	A	13^a^
F	R	M	T	G	11
L	R	M	S	G	8
F	S	M	S	G	6
L	R	M	T	A	5
F	R	M	T	A	3
F	R	M	N	A	3
F	R	M	S	G	3
F	S	M	T	A	3
L	R	M	S	A	2
F	S	T	N	G	2
F	S	M	S	A	1
L	S	M	T	G	1
L	S	T	S	A	1
F	R	T	N	A	1
L	R	T	N	G	1
F	S	T	T	A	1
L	S	M	T	A	1
F	S	M	T	G	1
L	R	M	T	G	1
F	S	M	N	A	1
F	R	M	N	G	1

Total isolates = 70; 69 isolates D0; 1 isolate DR

^a^For recurrence isolate, genotype day 0 = day 14

### Mutant alleles of the *pvdhps* gene

Analysis of the *pvdhps* gene of the 70 subjects revealed only one mutant allele, 383G in 35 subjects (50 %). The recurrent isolates at day 14 carried no mutant alleles of the *dhps* gene.

## Discussion

Analysis of the *pvdhfr* and *pvdhps* genes in *P. vivax* isolates from five different malaria-endemic areas in Indonesia revealed a wide distribution of the mutant alleles associated with resistance to SP. The mutant alleles of the *dhfr* gene were found either as single polymorphisms or in combination with other polymorphisms. With the *dhps* gene, 383G was the only mutant allele observed. Previous reports found similar polymorphisms in *P. vivax* isolates from Lampung and Papua [17, 18]. The findings indicate that the SP drug pressure to *P. vivax* may have taken place at all sites and that mistreatment of *P. vivax* malaria infections may be widespread. Until 2010, the recommended drug for vivax malaria was the combination of chloroquine with primaquine but due to the spread of chloroquine resistance to *P. falciparum* and *P. vivax*, ACT was introduced [24].

Three types of allelic combination of *pvdhfr* gene were observed among the *P. vivax* isolates from the five malaria-endemic areas investigated. These included double, triple and quadruple mutants. Overall, when looking at the allelic combinations, the frequency of double mutants was 22.6 % (36 isolates), triple mutants was 2.5 % (four isolates) and quadruple mutants was 15.1 % (24 isolates). *Plasmodium vivax* isolates from Papua were dominated by quadruple mutants (61.8 %). Nevertheless, the in vivo study in Sumba to determine the molecular basis of SP treatment failure in *P. vivax* indicated that all *P. vivax* isolates that carried the aforementioned allelic combination were still susceptible to SP, except in one recurrent isolate at day 14 with the double *dhfr* mutants, 58R/61M.

Mutations in *pvdhfr and dhps* genes, including 58R and 117N, have been implicated in pyrimethamine and sulfadoxine resistance, respectively [25]. In *P. falciparum*, the existence of quintuple mutations, three in *dhfr* gene (S118N, C59R and N51C) and two in *dhps* gene (A437G and K540E), have been associated with SP treatment failure [26–28]. The corresponding mutations in *P. vivax* are 117N, 58R and 49R in *dhfr* gene and 383G and 553G in *dhps* gene. Although *dhfr* mutations have been widespread among the *P. vivax* isolates, mutations at the *dhps* gene are still rare. The 383G allele was found in various frequency among the isolates examined and the highest frequency was observed in Purworejo.

Two and three GGDN repeat units were detected in wild and mutant types *dhfr* in all allelic combination. This result corroborates the previous results where an insertion/deletion event within the short repetitive region did not appear to be clearly associated with antifolate resistance. However, repetitive sequences that produce length polymorphism may not affect pyrimethamine sensitivity [24].

The wide distribution of *dhfr* and *dhps* gene polymorphisms among the *P. vivax* field isolates seems to have little implication on the efficacy of SP combination treatment. This was demonstrated in the in vivo study in west Sumba District, where the efficacy of SP was still very high (94 %). This is the first report from Indonesia that demonstrates that *P. vivax* is highly sensitive to SP. The results of this study however require further observation in areas where SP was reported to be resistant to *P. falciparum*, such as Papua [18]. Unfortunately, with current policy that adopted ACT as first-line therapy for both *P. vivax* and *P. falciparum*, it will be difficult to justify a study to monitor the efficacy of SP anymore. Nevertheless, SP may be considered as a rational option for the treatment of vivax malaria either given alone or in combination with artemisinin. Previous studies in Pakistan and Afghanistan also found high efficacy of SP in the treatment of vivax malaria [29].

Most subjects carried triple and quadruple mutations in the *dhfr* gene in addition to the mutaion in *dhps* 383G, but still were sensitive to the SP. The only subject who showed late treatment failure carried the double mutations in *dhfr* without any *dhps* gene mutation. Here, it is not clear as to whether this parasite originated from new infection or was a recurrence from the current infection, keeping in mind that previous results indicate that hypnozoites may have a different genotype than that of the initial infection [30, 31]. Nonetheless, as SP has a long half-life, any parasite that was detected during the monitored 42-day treatment should be relatively resistant to SP. Previous results from Thailand also show inconclusive results upon the association between the *dhfr* and *dhps* mutations to the SP treatment outcome. Although it is clear that the isolates that carry multiple mutations in *dhfr* and *dhps* are associated with high grade SP resistance, many of the isolates still respond adequately to the SP [23]. In this regard, it is important to note that in Indonesia, the proportion of *P. vivax* isolates that carry double mutant *dhps* is still very rare and in fact was only reported in the isolates collected from northeastern Papua. Papua, with the highest amount of malaria and consequent highest selection pressure from mistreatment of vivax infections with SP, may explain this high number of multiple resistant alleles. These results may explain why the *P. vivax* isolates in Sumba are still susceptible to SP and this also may be true of the isolates from Lampung, Purworejo, Mataram and, to a lesser extent, in Papua.

The development and spread of drug-resistant parasite strains is a major obstacle to the malaria control and elimination programme. As the molecular basis of the parasite resistance to antifolates and sulfa drugs has been well established in *Plasmodium* spp., analysis on the frequency distribution of *dhfr* and *dhps* mutant alleles would provide a better perspective on the use of SP in a particular area. In this regard, it is important to notice an increasing prevalence of *P. vivax* isolates carrying the triple mutants of *dhfr* gene in all sites and particularly the quadruple mutants of *dhfr* gene in northeastern Papua regions.

## Conclusions

This study revealed a wide distribution of *dhfr* and *dhps* mutant alleles among *P. vivax* isolates across broad geographic regions of Indonesia, but an impact on the SP efficacy was not observed at this point. Therefore, with proper malaria diagnosis, SP may still be used as a rational anti-malarial drug, either as a single prescription or in combination with artemisinin.
